# Isotherm and Kinetic Modeling of Strontium Adsorption on Graphene Oxide

**DOI:** 10.3390/nano11112780

**Published:** 2021-10-20

**Authors:** Abdulrahman Abu-Nada, Ahmed Abdala, Gordon McKay

**Affiliations:** 1 Division of Sustainable Development, College of Science and Engineering, Hamad Bin Khalifa University, Education City, Qatar Foundation, Doha 34110, Qatar; aabunada@hbku.edu.qa; 2 Chemical Engineering Program, Texas A&M University at Qatar, Education City, Doha 23874, Qatar; ahmed.abdala@qatar.tamu.edu

**Keywords:** graphene oxide, adsorption, strontium, adsorption isotherms, adsorption kinetics

## Abstract

In this study, graphene oxide (GO) was synthesized using Hummers method. The synthesized GO was characterized using field-emission scanning electron microscopy (FE-SEM), X-ray diffraction (XRD), Fourier transformed infrared (FTIR) spectroscopy, X-ray photoelectron spectroscopy (XPS), and Brunauer–Emmett–Teller (BET) nitrogen adsorption. The analyses confirmed the presence of oxygen functional groups (C=O and C-O-C) on the GO surface. These oxygen functional groups act as active sites in the adsorption Sr (II). The BET analysis revealed the surface area of GO of 232 m^2^/g with a pore volume of 0.40 cm^3^/g. The synthesized GO was used as an adsorbent for removing Sr (II) from aqueous solutions. The adsorption equilibrium and kinetic results were consistent with the Langmuir isotherm model and the pseudo-second-order kinetic model. A maximum strontium adsorption capacity of 131.4 mg/g was achieved. The results show that the GO has an excellent adsorption capability for removing Sr (II) from aqueous solutions and potential use in wastewater treatment applications.

## 1. Introduction

The discharge of effluents from natural and artificial sources relating to industrial activities, such as electroplating, metal smelting, drilling, and the production of oil and gas, can pollute wastewater with heavy metals, leading to adverse effects on health and the environment. Technologies such as membrane filtration systems, coagulation, ion exchange, and adsorption are used as treatment methods to remove such metals [1]. Although some technologies have proven effective, most of these technologies are costly and produce additional discharge streams [2]. However, adsorption can be one of the less costly treatment methods due to the unique nature of the removal process in which adsorption occurs; there is less permanent bonding between the adsorbent and the pollutant [3]. This phenomenon can be utilized in regenerating and recycling the adsorbent, thus decreasing the cost of the overall treatment process.

Graphene is a single layered material with a unique two-dimensional structure which is made up of sp^2^ bonded carbon atoms. It is one of the heavily researched two-dimensional adsorbents along others like molybdenum disulphide and platinium doped Tungsten diselenide [4,5]. Recently, there has been significant research on the development of graphene-based adsorbents to remove heavy metals. Guo et al. (2014) synthesized an amino-functionalized magnetic graphene composite to remove lead, mercury, cadmium, and chromium ions from their aqueous solutions [6]. Shahzad et al. (2017) used EDTA-functionalized chitosan-graphene oxide (GO) nanocomposite to adsorb lead, copper, and arsenic from water and achieved adsorption capacities of 206, 207, and 43 mg/g, respectively [7]. Wang et al. (2013) used GO to remove zinc from aqueous solutions and reported a maximum capacity of 246 mg/g [8]. Raghubanshi et al. (2017) synthesized graphene oxide and used it to remove lead ions from aqueous solutions with a maximum capacity of 120 mg/g [9]. Abu Nada et al. (2020) reviewed the publications on the applications of hybrid graphene composites for metals removal from wastewater [10].

The adsorption of strontium has only received limited attention despite its presence in produced waters from oil exploration drilling and its application in flares and fireworks, paints and plastics, and medicine [11]. Jang et al. (2018) synthesized a three-dimensional barium-sulfate-impregnated reduced GO (rGO) aerogel using a self-assembling hydrothermal method to remove strontium ions from aqueous solutions and achieved an adsorption capacity of 233 mg/g [12]. Khalil et al. (2017) used EDTA functionalized GO to remove cobalt and strontium from an aqueous solution with a capacity reaching 197 and 158 mg/g for cobalt and strontium, respectively [13]. Xing et al. (2019) studied the adsorptive removal of strontium ions from an aqueous solution by GO, and the maximum adsorption capacity was 138 mg/g. However, they did not study the kinetics of the adsorption process [14]. Wen et al. (2014) used GO-hydroxyapatite nanocomposite to capture Sr ions from aqueous solutions, and an excellent adsorption capacity of 702 mg/g was obtained [15]. Minitha et al. (2018) applied magnetite nanoparticles decorated rGO composite to remove cesium and strontium ions from aqueous solutions with a maximum capacity of 128 and 385 mg/g, respectively [16].

This study investigates the adsorption of strontium ions onto GO. Different characterization methods were applied to study the adsorbent properties. The characterization tests describe the structure, crystallinity, chemical composition, surface area, and surface functional groups availability on GO. The GO adsorbent was tested in adsorption experiments under various conditions and using different parameters to study the strontium adsorption performance. The experimental data were correlated by the kinetic and isotherm models to determine which model best describes the adsorption mechanisms. The novelty of this study is the extensive kinetic modeling of the adsorption of strontium onto GO to further understand the adsorption process mechanism.

## 2. Materials and Experimental Methods

### 2.1. Materials

Strontium chloride hexahydrate (SrCl_2_·6H_2_O) (Sigma Aldrich, St. Louis, MI, USA) and nitric acid (HNO_3_, 15.7 M) (Sigma Aldrich, St. Louis, MI, USA) were used to prepare a standard strontium solution (50 mg/L). A stock solution of 1.0 g/L Sr^2+^ was prepared by dissolving strontium chloride hexahydrate (SrCl_2_·6H_2_O) in deionized water. Natural flake graphite (XFNano, Nanjing City, China, 99% purity), sulfuric acid (Fisher Chemical, Hampton, NH, USA, 95%), and potassium permanganate (Sigma Aldrich, St. Louis, MI, USA), sodium nitrate (Sigma Aldrich, St. Louis, MI, USA), and hydrogen peroxide (Sigma Aldrich, St. Louis, MI, USA, 30%) were used to prepare graphite oxide.

### 2.2. GO Synthesis

GO was synthesized using the Hummers method [17]. Briefly, H_2_SO_4_ (40 mL), graphite (1 g), and NaNO_3_ (1 g) were mixed in a 500 mL round bottom flask. KMnO_4_ (6 g) was then slowly added under stirring at 35 °C. The solution was kept at 35 °C for 1 h. Then Milli-Q water (80 mL) was added, and the solution was stirred for 30 min at 90 °C. H_2_O_2_ (6 mL) and Milli-Q water (150 mL) were slowly added to the solution. The mixture was then filtered, and the solid GO was collected and washed using deionized water, followed by centrifugation at 10,000 rpm and decanting of the supernatant. The washing process was repeated multiple times until the pH reached 5.8. The collected GO was suspended in water, sonicated, and freeze-dried.

### 2.3. GO Characterization

The presence of functional groups on the GO surface was analyzed using X-ray photoelectron spectroscopy (XPS) (Thermo Fisher, ESCALAB, 250Xi, Waltham, MA, USA). FTIR spectroscopy (Thermo Scientific Nicolet iS50, Waltham, MA, USA) was also used to study the chemistry of the GO surface. The FTIR spectrum was collected at a 4 cm^−1^ spectral resolution and 64 scans. The sample was crushed with KBr in a mortar at a ratio of 1:100, and the pressed pellet was immediately analyzed in the region of 400–4000 cm^−1^. The GO crystal structure was analyzed using powder XRD (Rigaku SmartLab, Akishima, Tokyo, Japan). The thickness of the GO sheets was measured using AFM (Bruker Icon Dimension Atomic Force Microscope, Cambridge, England). The surface area, porosity, and pore size distribution were measured using N_2_ adsorption at −77 K (Micromeritics ASAP 2020 Plus, Atlanta, GA, USA). The sample was degassed at 85 °C for 1 h prior to the adsorption measurements to remove any adsorbed moisture.

The thermal stability of GO was analyzed using TGA (Discovery SDT-650, New Castle, DE, USA) under a nitrogen environment at a heating rate of 5°/min. The morphology of GO sheets was analyzed via scanning electron microscopy (SEM) (JEOL 7610F, Akishima, Tokyo, Japan).

### 2.4. Adsorption Measurements

A 1.0 g/L Sr^2+^ stock solution was prepared using strontium chloride hexahydrate salt. The solution was then used to prepare 50 mL samples with initial concentrations ranging between 20 and 215 mg/L. The required GO dose was added to the Sr^2+^, and the solution was agitated at 25 °C for 120 min using a magnetic mixer at 500 rpm. The strontium concentration was measured using inductively coupled plasma optical emission spectrometry (IPC-OES) (Agilent Technologies 5110 ICP-OES, Santa Clara, CA, USA). The sample was centrifuged for 5 min, run through a 0.22 µm filter, and diluted to concentrations not greater than 10 ppm before performing the concentration measurements. The sample concentration was calculated using a calibration curve made using a Sr standard in the range of 0–10 ppm. The kinetic experiments were performed by shaking a 100 mL strontium chloride solution with concentrations of 20 to 215 mg/L containing a fixed GO dose. Samples were taken after different durations, and the Sr concentration was measured. The optimum pH was determined by carrying the adsorption at pH ranging from 3 to 11, adjusted using 0.1 M HCl and 0.1 M NaOH.

The adsorption capacity, q_t_, at a specific time t and the removal percentages were calculated as follows:

(1) $$q_{t}=\frac{\left(C_{0}-C_{t}\right)V}{W}$$

(2) $$\mathrm{Removal}\%=\frac{\left(C_{o}-C_{t}\right)}{C_{o}}\times 100$$

where C_o_ (mg/L) and C_t_ (mg/L) are the initial and equilibrium solute concentrations, V (mL) is the volume of the solution, and W (mg) is the weight of the adsorbents. All experimental data were averages of triplicate samples with the error being ±5%.

## 3. Results and Discussion

### 3.1. GO Characterization

**Figure 1:**
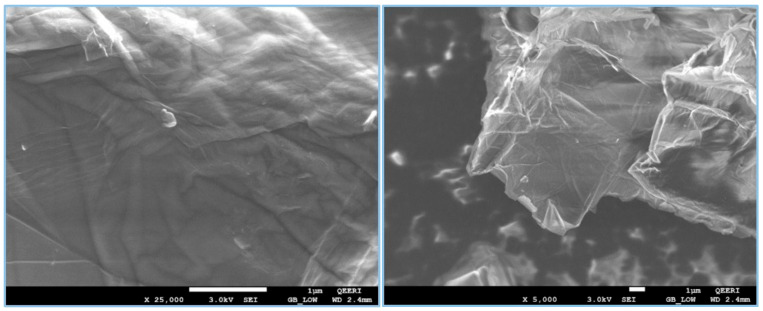
SEM images of GO showing the sheet-like morphology.

The morphology of the synthesized GO was analyzed using SEM. As shown in Figure 1, the adsorbent comprises large particles with irregular shapes resembling flakes or sheet-like structures with wrinkled surfaces. Multiple samples were tested with the sheet dimensions ranging between 500 nm and 5 µm.

XPS was used to analyze the elemental composition and bonding at the surface of the adsorbent. An X-ray beam is emitted at the sample under vacuum conditions, and the kinetic energy of the escaped electrons was measured [18].

**Figure 2:**
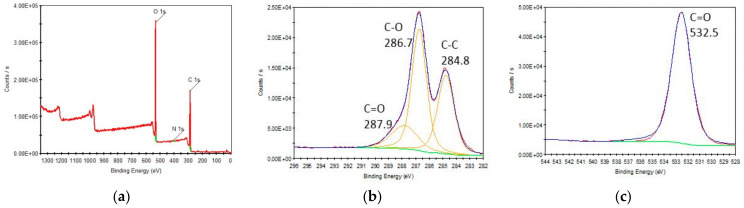
XPS (**a**) survey and deconvoluted (**b**) C1s, and (**c**) O1s spectra.

The result of the X-ray photoelectron spectroscopy analysis on GO is shown in Figure 2 and Table 1 shows the elemental breakdown of the adsorbent. The oxygen content is 28–37%, and the carbon content is 61–72%. The core-level spectra are taken at 20 eV pass energy with a step of 0.1 eV. The survey spectrum (5–1350 eV) shows the Mn, O, and C signals. The manganese present is residual from the oxidizing agent used to synthesize the adsorbent (potassium permanganate). It also shows a typical graphene oxide XPS spectrum with C-C is located at 284.8 eV, a strong C-O at 286.7 eV, and a low-intensity C=O at 287.9 eV.

X-ray diffraction is used to determine the crystallinity of the adsorbent by measuring their diffraction pattern. It is also used to identify the crystalline structure of a mixture of multiple microcrystalline materials. When the X-ray hits the material, a secondary diffracted beam is bounced off it, reading the inter-planar spacing of the crystalline structure, namely the d-spacing [19].

**Figure 3:**
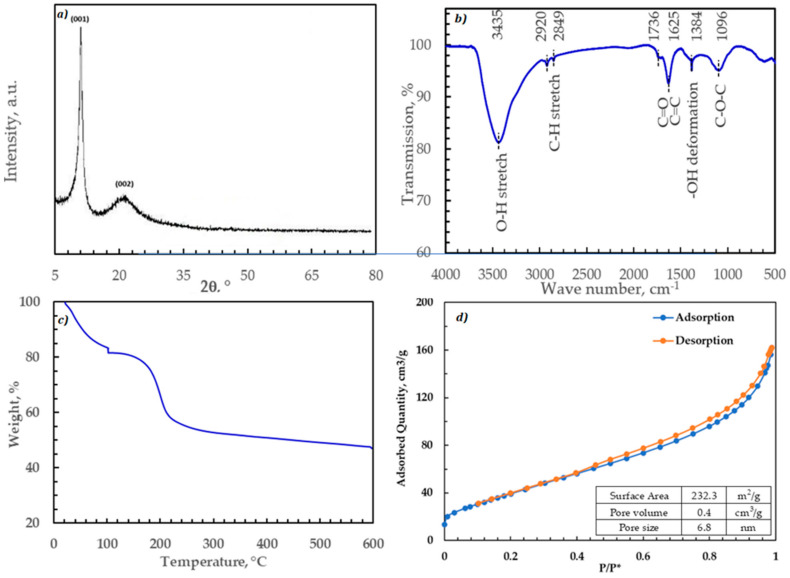
Characterization figures for the GO used in this study: (**a**) XRD X-ray diffraction pattern; (**b**) FTIR analysis; (**c**) TGA thermal stability curve; (**d**) BET nitrogen hysteresis curves for GO at 77 K.

The XRD diffraction pattern of GO is shown in Figure 3a. The typical GO diffraction peaks are observed with the diffraction peak at 2θ = 11.1°, corresponding to the (001) plane with d-spacing of 7.96 Å. The GO crystallite size (τ) was 79.5 nm, as determined using the Scherrer equation using the Scherrer formula [20]. The mean crystallite length in the C direction was also found using Scherrer’s formula to be 15.8 nm. Then, the number of layers was determined by dividing the mean crystallite length by the d-spacing to be ~21 layers. These results were consistent with other studies performed on GO [21,22].

The FTIR spectrum shown in Figure 3b of GO is typical. The characteristic features in the FTIR spectrum of GO are C=O (carbonyl/carboxy 1725 cm^−1^), C=C (aromatics 1632 cm^−1^) and C-O-C (epoxide 1093 cm^−1^). The broad peak at 3436 cm^−1^ is assigned to the hydroxyl groups (-OH).

The thermal stability of GO is analyzed using TGA. This analysis is important to investigate the applicability of thermal regeneration of the adsorbed strontium. It is also used to determine the maximum degassing temperature during the BET analysis without altering the adsorbent. The results showed that the moisture content of the adsorbent was almost 15%. It also reveals that GO is thermally stable until 160 °C. Above 160 °C, the degradation rate increased till 215 °C due to the loss of oxygen functionality, then decreased significantly.

The surface area, pore size, and pore size distribution play a significant role in the performance of adsorbents. The GO sample was analyzed via N_2_ adsorption at 77 K and the surface area was determined using the Brunauer, Emmett, and Teller (BET) method [21]. The pore volume and pore size distribution were calculated from the adsorption step using the Barret–Joyner–Halenda (BJH) method.

### 3.2. Adsorption Studies

The application of graphene oxide in water spiked with strontium was performed. The influence of adsorbent dosage, contact time, pH, and initial contaminant concentration were studied.

#### 3.2.1. Effect of GO Dosage

**Figure 4:**
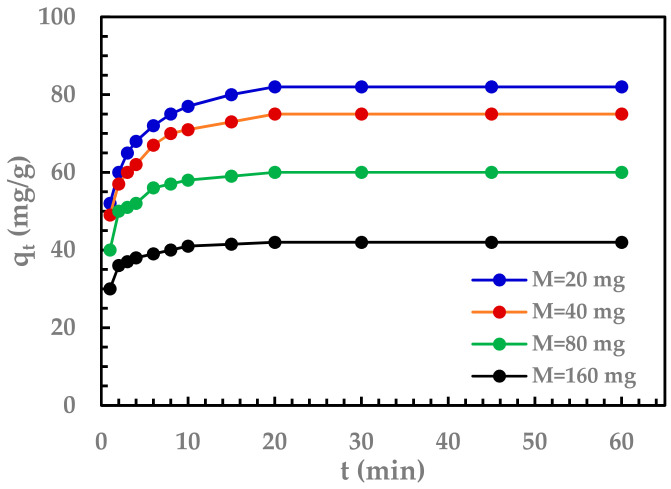
Effect of GO dosage on the capacity for removal of Sr (II). Initial Sr (II) concentration: 150 mg/L, volume = 100 mL, agitation using magnetic stirrer 500 rpm at 25 °C, initial pH 5.

The Sr (II) adsorption was examined at different dosages of GO (0.2, 0.4, 0.8, and 1.6 g/L), and the rate curves are presented in Figure 4. It is evident that with increasing adsorbent dosage, the adsorption capacity decreases due to the reduction in the adsorbate (Sr^2+^) to adsorbent (GO) ratio.

#### 3.2.2. Effect of pH

**Figure 5:**
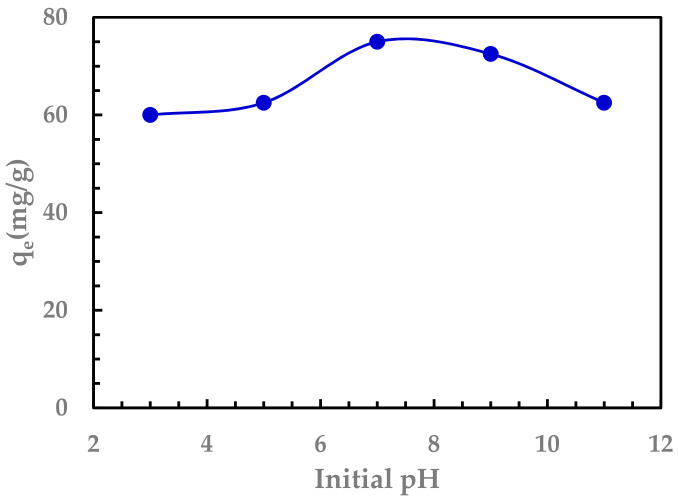
Effect of initial pH on the capacity for Sr (II). Initial Sr (II) concentration: 200 mg/L, volume = 50 mL, agitation using magnetic stirrer 500 rpm at 25 °C, adsorbent dosage 10 mg.

The effect of initial pH on the adsorption process was analyzed by carrying out the batch adsorption experiment at pH 3, 5, 7, 9, and 11. The Sr adsorption capacities are above 60 mg/g in the pH range from 3 to 11, as shown in Figure 5. The optimum pH was found to be 7. At this pH, a mass dosage of 10 mg and an initial concentration of 200 mg/L, the equilibrium adsorption capacity was 75 mg/g.

### 3.3. Adsorption Isotherm

**Figure 6:**
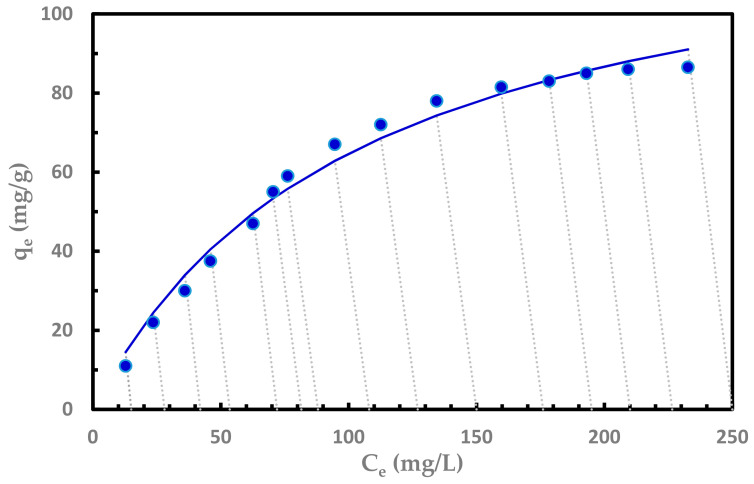
Langmuir isotherm model fitted to the adsorption data of Sr (II) with variable initial concentrations on GO with 10 mg and volume of 50 mL, initial pH 5, and 25 °C.

Multiple adsorption equilibrium experiments were carried with different initial Sr^2+^ concentrations. Results were fitted to Langmuir [22], Freundlich [22,23], Redlich-Peterson [24], Sips [25], Temkin [26], and Toth [27] isotherm models. The Langmuir isotherm model provides the best fitting to the adsorption of Sr^2+^ on GO, as shown in Figure 6, where the dotted lines represent the system operating lines with a −volume/mass slope.

The model constants for the six isotherm models are presented in Table 2 with the error analyses. The Langmuir SSR value is the lowest, but the SSR for the Redlich-Peterson, Sips, and Toth is also very close. The exponent of these models was 0.99, i.e., very close to 1.00. Since an exponent of 1.00, reduces these isotherm models to the Langmuir isotherm, the adsorption process is a Langmuir type adsorption process. The Langmuir isotherm is based on monolayer adsorption on the active sites of the adsorbent surface. The nonlinear form of the Langmuir isotherm is expressed in Equation (3) as follows:

(3) $$q_{e}=\frac{K_{L}C_{e}}{1+a_{L}C_{e}}$$

where q_e_ and C_e_ are the amount of the adsorbate adsorbed per unit mass of adsorbent material (mg/g) and the equilibrium concentration of adsorbate (mg/L), respectively. K_L_ and a_L_ are Langmuir constants representing the monolayer capacity and equilibrium constant, respectively. A fundamental characteristic of the Langmuir model is the dimensionless constant (R_L_), generally known as the separation factor, represented as:

(4) $$R_{L}=\frac{1}{1+a_{L}C_{o}}$$

where C_o_ is the highest initial concentration (mg/L). The value of R_L_ indicates whether adsorption is irreversible (R_L_ = 0), favorable (0< R_L_ < 1), linear (R_L_ = 1), or unfavorable (R_L_ > 1) [28]. In this study, R_L_ = 0.097 shows very favorable adsorption.

### 3.4. Adsorption Kinetics

It is essential to study the adsorption kinetics in wastewater treatment, whether it is to evaluate a new adsorbent or to remove a particular pollutant. The kinetics also provide an insight regarding the adsorption mechanism and provide information on the uptake rate. The uptake rate, which can be used to accurately predict the contact time based on the parameters from the kinetic models, is used in the design of wastewater treatment plants to estimate the residence time and based on this value, the optimum size of the plant and equipment for minimum CAPEX [29]. Several kinetic models have been correlated with the experimental data; each model is based on different mechanistic assumptions, and these models are Avrami [30], the pseudo-second-order [31,32], Elovich [33,34], and the intraparticle diffusion model [35]. The equation of each model and the fitting results are presented in Table 3.

**Figure 7:**
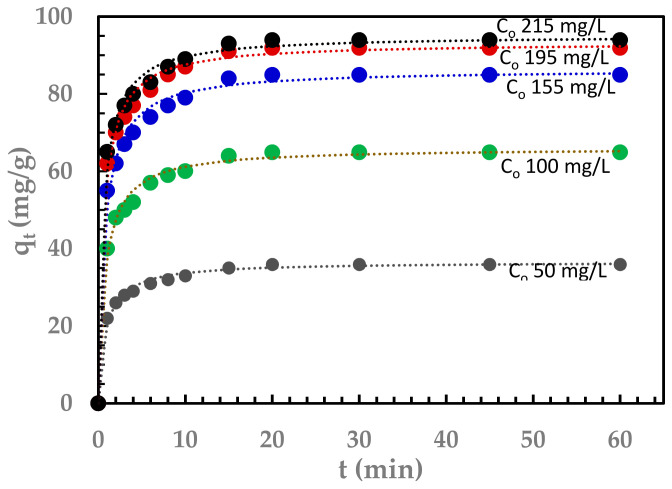
PSO model fitted to adsorption of five initial concentrations of Sr. GO dose = 20 mg, liquid volume = 100 mL, pH = 5, and T = 25 °C.

The fitting of the experimental results using the pseudo-second-order model is shown in Figure 7. The experiments were conducted at various initial Sr (II) concentrations, a constant GO adsorbent dosage of 20 mg, and pH = 5. Aliquot samples were taken out for analysis multiple times. The results were used to determine the adsorption kinetics, which describes the effect of contact time on the uptake of Sr (II) and the time it takes for the reaction to reach equilibrium. All other parameters were kept constant at initial pH = 5. Around 90% of the adsorption takes place rapidly during the initial 15 min. Then the adsorption follows at a slower rate. This high initial rate of adsorption has benefits in the process design of the wastewater treatment plants. Reducing the required residence time ultimately reduces the equipment size and consequently the CAPEX and OPEX expenditures.

The pseudo-second-order model was developed by Ho and McKay (1999). The driving force for the adsorption is the difference between equilibrium adsorption capacity (q_e_, mg/g) and the adsorbed capacity at time t (q_t_, mg/g). k_2_ is the rate constant. However, the adsorption rate is proportional to the square of the driving force, indicating each adsorbate ion occupies two adsorption sites [31].

The differential form of the model is shown in Equation (5):

(5) $$\frac{{\mathrm{dq}}_{t}}{\mathrm{dt}}=k_{2}{\left(q_{e}-q_{t}\right)}^{2}$$

The integral form of the model is given by Equation (6):

(6) $$q_{t}=\frac{q_{e}^{2}k_{2}t}{1+q_{e}k_{2}t}$$

The adsorption capacities for different initial concentrations of the strontium adsorbate were determined. As expected, the higher the initial strontium concentration, the higher the adsorption capacity due to the increasing availability of Sr (II) ions as the concentration increases, thus occupying more of the available active sites.

Table 4 shows the comparison between a range of various adsorbents for removing strontium from aqueous solutions and their respective capacities and isotherm models. The GO displayed excellent sorption capacity for Sr (II). Moreover, the functionalization of GO can further increase its adsorption performance as a backbone adsorbent for deployment in practical water treatment applications [28].

## 4. Conclusions

In this study, GO was synthesized by Hummer’s method, which involves the chemical oxidation of graphene. The morphology and structure of the adsorbent were characterized by SEM, FTIR, XRD, XPS, BET, and TGA. GO showed a good adsorption capacity of Sr (II). The Langmuir model well describes the equilibrium adsorption isotherms. The maximum adsorption capacity at 25 °C was 131.4 mg/g. The kinetics analysis revealed the adsorption process follows the pseudo-second-order kinetic model, with a rapid equilibrium time of ~20 min. These results suggest GO could be an excellent adsorbent for efficiently removing Sr (II) from an aqueous solution.

## Figures and Tables

**Table 1:** The atomic ratios of the elements present in the adsorbent.

Name	Peak BE	FWHM eV	Area (P) CPS.eV	Atomic %
C 1s	258.99	4.24	691,735.15	62.15
O 1s	532.22	3.16	1,045,248.53	36.98

**Table 2:** Parameters of the different isotherm models.

Isotherm Model	Equation	Model Parameters	Fitting Quality
Langmuir	$q_{e}=\frac{K_{L}C_{e}}{1+a_{L}C_{e}}$	K_L_ = 1.274 L/mg a_L_ = 0.0097 q_max_ = 131.41	SSR = 133.4 R^2^ = 0.985
Freundlich	$q_{e}=a_{F}C_{e}^{b_{F}}$	a_F_ = 5.359 b_F_ = 0.529	SSR = 492.0 R^2^ = 0.945
Redlich-Peterson	$q_{e}=\frac{K_{R}C_{e}}{1+a_{R}C_{e}^{b_{R}}},\;0<b_{R}<1$	K_R_ = 1.274 a_R_ = 0.0097 b_R_ = 0.999	SSR = 134.3 R^2^ = 0.985
Sips	$q_{e}=\frac{K_{\mathrm{LF}}C_{e}^{n_{\mathrm{LF}}}}{1+a_{\mathrm{LF}}C_{e}^{n_{\mathrm{LF}}}},\;0<n_{\mathrm{LF}}<1$	K_LF_ = 1.27 a_LF_ = 0.0097 n_LF_ = 0.999	SSR = 134.1 R^2^ = 0.985
Temkin	$q_{e}={\mathrm{BlnA}}_{T}+{\mathrm{BlnC}}_{e}$	B = 29.32 A_T_ = 0.0930	SSR = 172.2 R^2^ = 0.981
Toth	qe=qmCe(KT+Cen)1n, 0<n<1	K_T_ = 103.16 n = 0.999 q_max_ = 131.40	SSR = 134.0 R^2^ = 0.985

**Table 3:** Summary of the parameters for each kinetic model fitted.

Kinetic Model	Equation	Model Parameters	Fitting Quality
Avrami (n = 1)	$\frac{{\mathrm{dq}}_{t}}{\mathrm{dt}}=k_{1}\left(q_{e}-q_{t}\right)$	q_e_ = 11.474 mg/g k_1_ = −0.00177	R^2^ = 0.727 SSR = 14.621
Pseudo-Second Order	$\frac{{\mathrm{dq}}_{t}}{\mathrm{dt}}=k_{2}{\left(q_{e}-q_{t}\right)}^{2}$	q_e_ = 66.050 mg/g k_2_ = 0.189	R^2^ = 0.999 SSR = 1.59 × 10^−4^
Elovich	$q_{t}=\frac{1}{\beta }\ln\left(1+\alpha \beta t\right)$	α = 6952.490 β = 0.160	R^2^ = 0.929 SSR = 70.122
Intra-Particle Diffusion	$q_{t}=K_{\mathrm{IP}}*t^{0.5}+C$	K_IP_ = 3.226 C = 45.977	R^2^ = 0.769 SSR = 229.278
Avrami	$q_{t}=q_{e}\left(1-e^{-K_{\mathrm{av}}*t^{n_{\mathrm{av}}}}\right)$	q_e_ = 66.185 mg/g K_av_ = 0.915 n_AV_ =0.427	R^2^ = 0.991 SSR = 9.422

**Table 4:** Adsorbent isotherm models fitted and capacities for Sr (II) adsorption.

Adsorbent	Maximum Capacity (mg/g)	Isotherm Model	Ref
Activated Carbon	12.1	Freundlich	[36]
NSC@MS-4A	44.9	Freundlich	[37]
TiO_2_	70	Langmuir	[38]
Peanut Husk	38.0	Redlich-Peterson	[39]
AC	34.3	Generalized model	[40]
GO	131.4	Langmuir	This study

## Data Availability

The data presented in this study are available on request from the corresponding author.
